# Dynamic handedness inversion of self-organized helical superstructures enabled by novel thermally stable light-driven chiral hydrazone switches†

**DOI:** 10.1039/d4sc05007j

**Published:** 2024-09-10

**Authors:** Jingyu Chen, Zichen Wang, Yuexin Yu, Jun Huang, Xinyu Chen, Tongji Du, Xinyue Song, Haiyang Yuan, Shuai Zhou, Xiang-Guo Hu, Xingping Zeng, Shengliang Zhong, Ruochen Lan

**Affiliations:** a College of Chemistry and Materials, Jiangxi Normal University Nanchang 330022 China lanruochen@pku.edu.cn; b School of Materials Science and Engineering, Peking University Beijing 100871 China; c National Research Centre for Carbohydrate Synthesis, Jiangxi Normal University Nanchang 330022 China

## Abstract

Chiral hydrazone photoswitch features are its high thermal stability and negative photochromy, making it desirable in the fabrication of thermally stable optical device. However, chiral hydrazones capable of reversibly inversing chirality is scarcely reported. Herein, a series of new chiral hydrazone switches, HI-1, HI-2 and HI-3, were designed and synthesized. Due to the photoinduced configuration changes, the newly synthesized hydrazone photoswitch presents a surprising chirality inversion upon light stimulation. Photoisomerization of light-driven hydrazone switch molecules was investigated by nuclear magnetic resonance (NMR) spectra and Raman spectroscopy. The effect of the intramolecular hydrogen bond on photoresponsiveness was analyzed. By incorporating the photoswitch into a liquid crystal (LC) host, light-driven cholesteric liquid crystals (CLCs) with handedness invertibility, a feasible photonic bandgap tunability, and superior thermal stability were achieved. In addition, according to the optical-driven thermal stability of the hydrazone switches, the fine regulation of light-driven CLC materials with multistage photo stationary states was realized, and the application of CLC materials in erasable and rewritable display panels was also demonstrated.

## Abstract 2

Chiral hydrazone photoswitch features are its high thermal stability and negative photochromy, making it desirable in the fabrication of thermally stable optical device.

## Graphical abstract

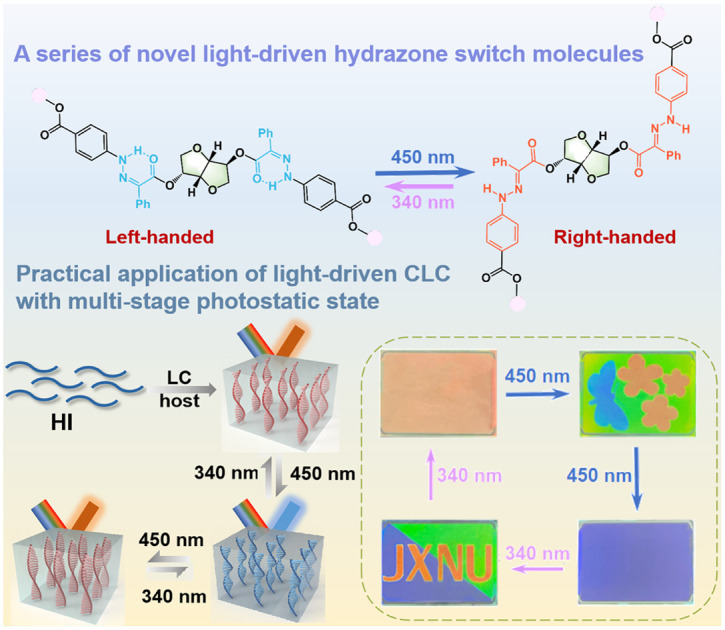

## Introduction

As a typical one-dimensional photonic crystal (PC), cholesteric liquid crystals (CLCs) are regarded as an emerging self-organized soft matter. The unique self-organized helical superstructure enables CLC to divide incident light into two circularly polarized lights (CPLs) and selectively reflect one of the CPL showing the same handedness as the CLC helix.^1–4^ CLC has been one of the promising candidates in the fabrication of chiroptical devices and chiral soft templates, and so on.^5–12^ According to the Bragg law, the central wavelength of the selective reflection band of CLC (*λ*) is determined by the average refractive index ***n*** and helical pitch (***p***) as: *λ* = ***n*** × ***p***. By regulating the length of the pitch to the scale of the wavelength of visible light, a vivid structural color of CLC can be observed due to the selective reflection of specific visible light. Besides, the sensitivity of the CLC to external stimulations makes it dynamic and performs reversible changes of optical properties stirred by light,^13–17^ heat,^18–21^ electricity^22^ and humidity.^23^ Ideally, using light to control the dynamic behavior of CLC is preferable because of the feasibility and incomparably high spatiotemporal precision, which can be achieved by incorporating a chiral photoswitch into the CLC matrix. In this case, the design and synthesis of the high-performance chiral photoswitch is the key to fabrication of advanced functional CLC materials.

In recent years, an abundance of chiral photoswitches has been put forward, including azobenzene,^24–28^ spiropyran,^29^ overcrowded alkene molecular motors,^30,31^ dithienylethene,^32^ unsaturated ketones and fulgides.^33^ By doping such chiral photoswitches into a CLC matrix, control of the pitch of the CLC helix as well as the shift of the reflection band can be judiciously realized. Particularly, a chiral photoswitch capable of inversing chirality is extremely promising due to the abilities to induce winding, unwinding and handedness inversion of the helical superstructure, endowing the CLC system capability to reflect both the left-handed and right-handed CPL and reversibly change the reflection band over an ultra-broad range.^34–37^ To meet the requirement of preparing high-performance CLC-based optical materials, a chiral photoswitch should have (1) a large helical twisting power (HTP) change and chirality inversibility to ensure wide-range tunability, (2) high thermal stability of the photo-induced state, and (3) slight absorption in the visible range to avoid the undesired interference of structural color and pigmented color.

A hydrazone photoswitch is a negative photochromic molecule showing a high thermal stability and large geometric changes, and has been considered as one of the most promising photoactive molecular machines.^3,24,38–43^ Bonding a chiral group onto the bulk of the hydrazone is a straightforward way to prepare a hydrazone-based chiral photoswitch.^44^ However, chirality invertible hydrazone-based chiral photoswitches are scarcely reported; our group reported the only example of a chirality invertible chiral hydrazone, which was prepared by linking the hydrazone group onto a binaphthol chiral core.^45^ Exploring novel hydrazone-based chiral photoswitches brings a great chance to combine the exceptional advantages of a hydrazone switch with CLC materials, which is hoped to provide an intriguing choice to prepare light-driven chiroptical photonic crystals and soft templates.

**Scheme 1:**
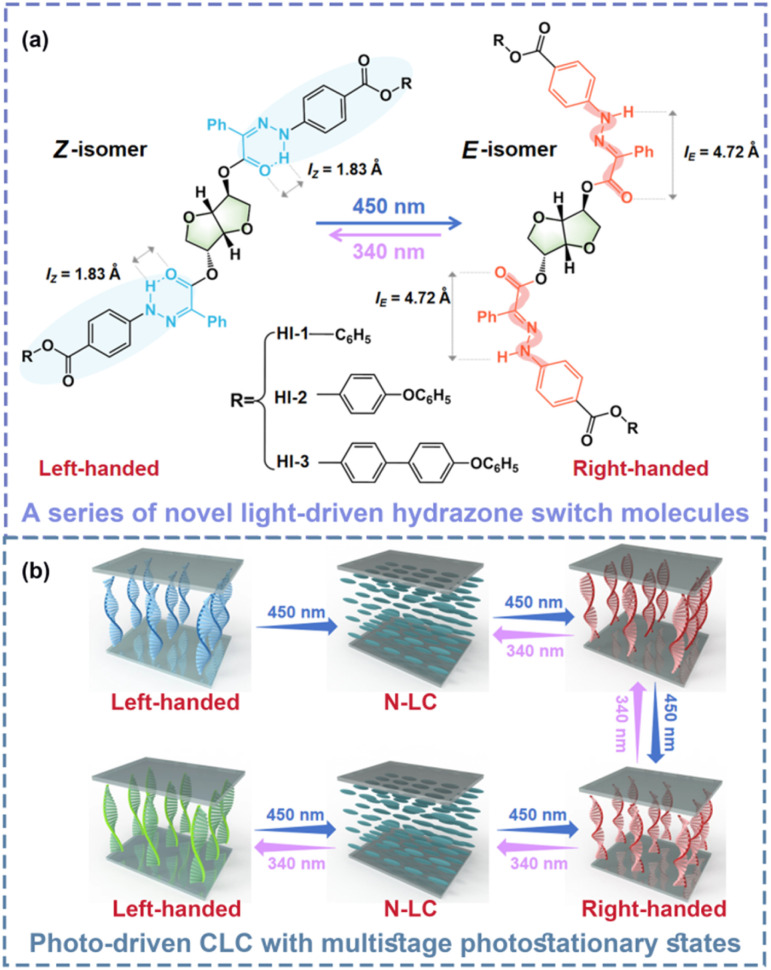
(a) Light-induced *Z*/*E* isomerization of HI-1, HI-2 and HI-3. (b) Schematic illustration of the pitch change and handedness inversion of a helical superstructure induced by light-driven HI-1, HI-2 and HI-3. N-LC: Nematic Liquid Crystal.

Herein, a series of novel chiral hydrazone molecules were designed and synthesized. Two hydrazone groups were linked directly to the central chiral part S-isosorbide (named as HI-1, HI-2 and HI-3, respectively). By 450 nm light or 340 nm light irradiation, the hydrazone group isomerizes from *Z* to *E* and from *E* to *Z*, respectively. The large geometric change of the molecular switch is hoped to transfer to the central chiral core to induce considerable chirality changes (Scheme 1a). Surprisingly, it was found that the chirality of such a photoswitch is reversibly invertible by alternating 450 nm light and 340 nm light stimulations, indicating a new chirality invertible photoswitch. By doping the novel chiral hydrazone into the LC matrix, a dynamic CLC with a photo-tunable photonic bandgap and handedness invertible ability can be judiciously prepared. More importantly, thanks to the bistable property of hydrazones, the photo-induced state of such a CLC is highly stable, enabling the CLC to sustain multiple photostationary states (PSS) (Scheme 1b). Besides, the negative photochromy of hydrazone presents nearly no pigmented color, facilitating the utilization in the fabrication of optical devices.

## Results and discussion

A series of chiral hydrazones with different end groups was synthesized. In our design, the rigid group and alkoxy group designed at the end promote the miscibility of hydrazone molecules with LC host. The concentration of these three hydrazone photoswitches can be up to 15–20 wt%. The three hydrazone switch molecules synthesized in this work all have a striking HTP change and chirality inversion ability. The as-synthesized HI-1, HI-2 and HI-3 photoswitches all possess a high HTP due to the large conjunction region based on the intramolecular hydrogen bond. Under 450 nm light irradiation, due to the excellent thermal stability of the hydrazone molecules, the pitch of the CLC can reach multi-stage photostatic states. In other words, by 450 nm visible light irradiation, configuration changes of HI led to the diminishing of π–π interactions, causing the HTP of the left-handed HI-1, HI-2 and HI-3 molecules to decrease drastically. Besides, isomerization of hydrazone molecules inverts the chirality of HI-1, HI-2 and HI-3. Further, the chirality of hydrazone molecules can reverse back to left-handed by 340 nm light stimulation. By introducing a new chiral hydrazone switch into LC, we realize the writing and erasing of arbitrary colorful patterns. The independent, fast-response display device has broad prospects in the field of display, circular polarization luminescence, anti-counterfeiting and encryption applications.

**Fig. 1:**
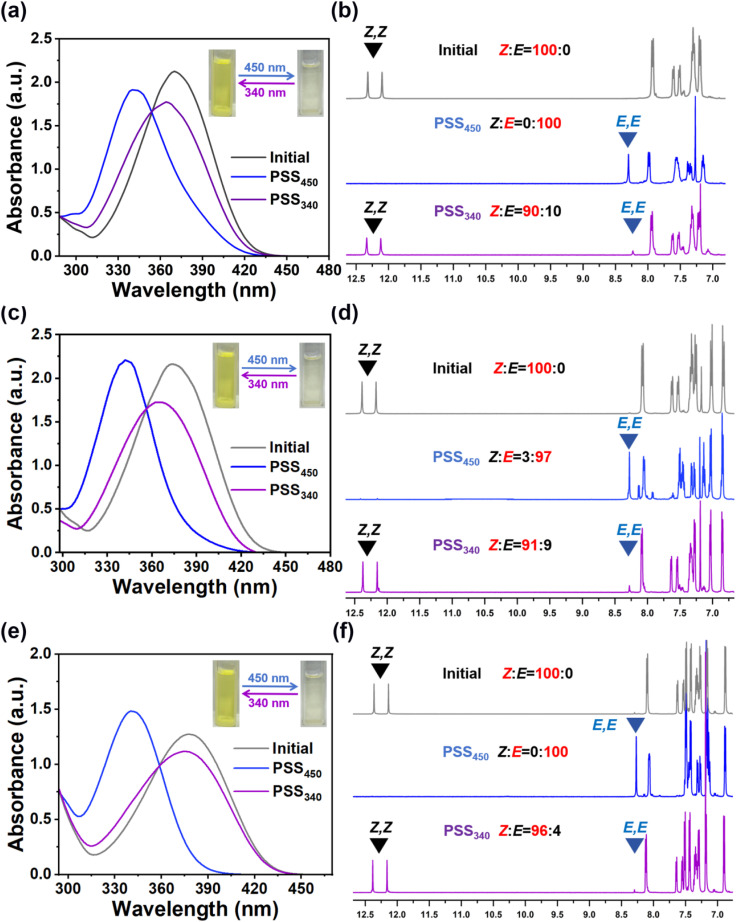
Absorption curve spectra of an EA solution of hydrazones (a) HI-1, (c) HI-2 and (e) HI-3 in different states. The concentration of hydrazone in EA was 10 μM. The insets show the appearance of the hydrazone solution under natural light. ^1^H NMR spectra of (b) HI-1, (d) HI-2 and (f) HI-3 in different states. The signals related to (*Z*, *Z*) and (*E*, *E*) isomers are noted by black and blue labels, respectively.

The detailed synthetic route and ^1^H, ^13^C NMR characterization results of HI-1, HI-2 and HI-3 are provided in the ESI.† The photoisomerization of HI-1, HI-2 and HI-3 was studied by recording the spectra of HI-1, HI-2 and HI-3 in different states using UV-vis spectroscopy. As shown in Fig. 1a, the absorption maxima of a blue-shift from 374 to 340 nm after 450 nm light irradiation (2 mW cm^−2^, 3 min), indicated that the HI-1 isomerizes to a photostationary state (PSS_450_). Subsequently, exposing the sample to 340 nm light (1 mW cm^−2^, 2 min) led the absorption peak to red-shift to 370 nm, reaching another stable state (PSS_340_). It can be seen from the illustration that after 450 nm light irradiation, the solution containing HI-1 changes from slight yellow to colorless, indicating that isomerization occurs during irradiation. Then, after 340 nm light irradiation, the yellow solution can be restored. By alternating the 450 nm and 340 nm light irradiation, HI-2 and HI-3 present similar photo-induced absorption and color changes. The photoresponsive behaviors of HI-1, HI-2 and HI-3 were then investigated in detail by ^1^H NMR spectroscopy. After 450 nm light irradiation for 600 s, the diminishing signal of the *Z* isomer and the appearance of a new signal related to the *E* isomer reveals a 100% conversion of HI-1 and HI-3 molecules to PSS_450_, as presented in Fig. 1d and f. In addition, 97% of the HI-2 was converted to the *E* isomer PSS_450_ (Fig. 1e). By 340 nm light exposure for 300 s, the signal related to the *Z* isomer reappears, while the signal referred to the *E* isomer remains, illustrating the incomplete reformation of the *Z* isomer, consistent with the results in Fig. 1a–c. By cycling tests, it was found that the absorption changes of HI-1, HI-2 and HI-3 have good fatigue resistance (Fig. S12–S14†).

**Fig. 2:**
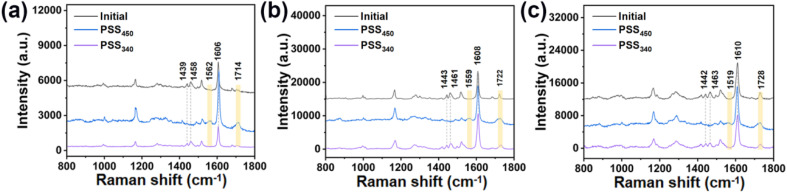
(a) Laser Raman spectra of HI-1 in different states. The light-yellow bars indicate the Raman peaks at 1439, and 1458 cm^−1^, which are characteristic of the *Z* isomer. The blue bars indicate the Raman peaks at 1562 and 1714 cm^−1^, which are characteristic of the *E* isomer. (b) Laser Raman spectra of HI-2 in different states. The light-yellow bars indicate the Raman peaks at 1443, and 1461 cm^−1^, which are characteristic of the *Z* isomer. The blue bars indicate the Raman peaks at 1559 and 1722 cm^−1^, which are characteristic of the *E* isomer. (c) Laser Raman spectra of HI-3 in different states. The light-yellow bars indicate the Raman peaks at 1442 and 1463 cm^−1^, which are characteristic of the *Z* isomer. The blue bars indicate the Raman peaks at 1519 and 1728 cm^−1^, which are characteristic of the *E* isomer.

The Raman measurement results of solid HI-1 were taken as an example to characterize the isomerization of chiral hydrazones (Fig. 2a). The bands at 1606 cm^−1^ correspond to the C=C stretching mode of the benzene. Upon isomerization, this peak does not shift. Therefore, the peak was chosen as a reference. After 450 nm light irradiation, the signals at 1439 and 1458 cm^−1^ disappear. Moreover, two new peaks appear at 1562 and 1714 cm^−1^. All these spectral changes indicate that the *Z* isomer undergoes a *Z* → *E* isomerization. The peak at 1606 cm^−1^ remains. Based on the intensity decrease of the bands at 1439 and 1458 cm^−1^, an almost 100% conversion from *Z* to *E* is achieved in the solid state after 450 nm light irradiation. Upon further irradiation with 340 nm light, the intensities of the bands at 1439 and 1458 cm^−1^ partially recover. Meanwhile, the intensities of the peaks at 1562 and 1714 cm^−1^ decrease. The results show that the three hydrazones undergo a consecutive isomerization process under 450 nm and 340 nm illumination.

**Fig. 3:**
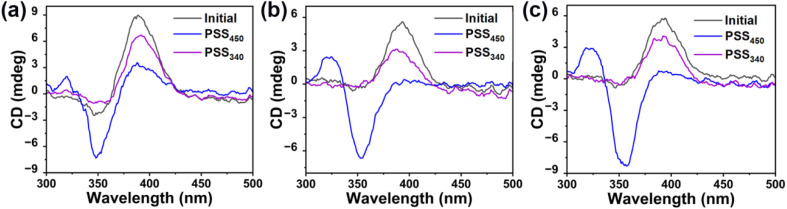
(a) CD spectra of HI-1 in the initial state, in PSS_450_ and in PSS_340_. (b) CD spectra of HI-2 in the initial state, in PSS_450_ and in PSS_340_. (c) CD spectra of HI-3 the initial state, in PSS_450_ and in PSS_340_.

Furthermore, the light-induced chirality changes of HI-1, HI-2 and HI-3 were measured using circular dichroism (CD) spectra. As shown in Fig. 3a–c, the as-synthesized HI-1, HI-2 and HI-3 present a positive absorption, indicating a left-handedness. Using 450 nm light to activate the HI-1, HI-2 and HI-3 into PSS_450_, the positive absorption band at 390 nm disappears and a new negative band occurs, indicating the chirality inversion of HI-1, HI-2 and HI-3 from left-handed to right-handed. When HI-1, HI-2 and HI-3 reach PSS_340_, the negative bands at 390 nm reappear, elucidating that the chirality of HI-1, HI-2 and HI-3 in PSS_340_ inverts to the initial left-handed state. Moreover, the signals assigned to the (*E*, *E*) isomer remain, illustrating the incomplete reformation of the (*Z*, *Z*) isomer, consistent with the results in Fig. 1.

**Fig. 4:**
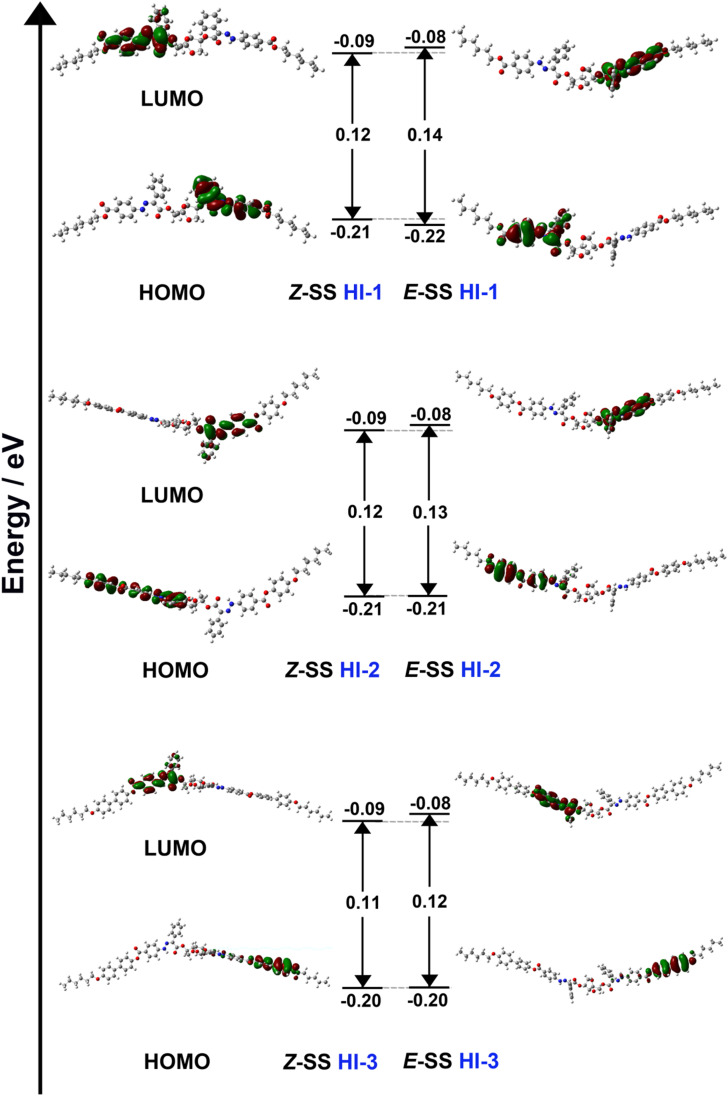
Energetic diagram and representation of HOMOs and LUMOs of the *E* and *Z* isomers of HI-1, HI-2 and HI-3 calculated using DFT (B3LYP/6-31G(d,p)).

From thermodynamic calculations, the energy difference between HOMO (highest occupied molecular orbital) and LUMO (lowest unoccupied molecular orbital) is called the band gap. The larger the difference in energy levels, the more energy the electrons need for the transition from HOMO to LUMO. The light energy absorbed by the hydrazone molecule can be stored in the *Z* state. The light energy stored in the *Z* state can be released as the spiral torsion force when isomerized to form the *E* state. As shown in Fig. 4, the band gap of HI-1, HI-2 and HI-3 in *E* is larger than that in *Z*. Therefore, it is possible to isomerize the hydrazone switches with low energy 450 nm light to obtain a nearly 100% high stability *E* isomer. To obtain the *Z* isomer, the *E* isomer can be irradiated by 340 nm light with a high energy level. However, due to the difference in stability, *Z* isomer cannot be completely recovered. Moreover, it was found that with the increase of the number of benzenes at the tail end, the comparison of free energy is as follows: HI-3 < HI-2 < HI-1 (Table 1). This indicates that HI-3 has the highest stability. The *Z* isomer can transform to the *E* configuration with 100% conversion by 450 nm light irradiation. At the same time, the HI-3 also has the largest *E* to *Z* conversion by 340 nm light irradiation. This is consistent with the ^1^H NMR results in Fig. 1d and e. The free energy, entropy and zero point energy of HI-1, HI-2 and HI-3 (Table 1), as well as the change of bond angle before and after isomerization of HI-1, HI-2 and HI-3, were calculated (Fig. S17†). The calculated results are consistent with the actual test results.

**Table 1:** Energy level calculations for HI-1, HI-2 and HI-3

Hydrazone	HI-1		HI-2		HI-3	
	*Z*-SS	*E*-SS	*Z*-SS	*E*-SS	*Z*-SS	*E*-SS
Δ*G* (J mol^−1^)	−2831.2237	−2831.2056	−3443.4288	−3443.4102	−3905.2913	−3905.2726
Δ*S* (J mol^−1^)	−2831.0510	−2831.0296	−3443.2253	−3443.2040	−3905.0660	−3905.0447
ZPE (J mol^−1^)	−2831.1113	−2831.0910	−3443.2972	−3443.2765	−3905.1472	−3905.1265

**Fig. 5:**
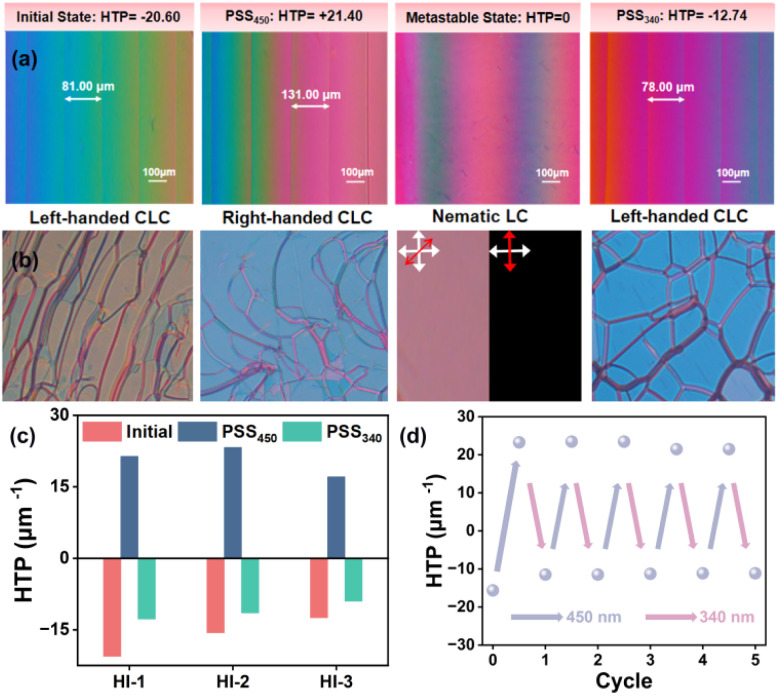
(a) Fingerprint texture of the light-responsive HI-1-doped CLC in different states. (b) Oil streak texture of the light-responsive HI-1-doped CLC in different states. The cross arrays represent the orthogonal polarizers of POM. (c) The diagram of HTP variations of three hydrazone switch molecules at the initial, PSS_450_ and PSS_340_ states. (d) Cycling test of the photo-induced HTP changes. The blue and purple arrays represent 450 nm and 340 nm light irradiation, respectively.

Taking hydrazone molecule HI-1 as an example, the Grandjean–Cano method was used to measure the HTP values of HI-1 in different states. As presented in Fig. 5a, the distance between adjacent disclination lines increased by 450 nm light irradiation until all of the disclination lines disappeared by continuous 450 nm light stimulation. Then, the disclination lines reappeared at PSS_450_, corresponding to the gradual unwinding and reformation of helices. According to the distance between disclination lines in different states, the HTP values of HI-1 were calculated. The pure (*E*, *E*) isomer presents an HTP of +21.40 μm^−1^ (mol%). After 340 nm irradiation, HTP can be restored to −12.74 μm^−1^. The light-driven handedness inversion of HI-1-doped CLC was further confirmed by filling the CLC into a planar cell. HTP tests related to HI-2 and HI-3 are shown in Fig. S15 and S16.† A typical oil streak texture could be observed by polarized optical microscopy (POM) (Fig. 5b). Using 450 nm light as a trigger, the texture of the CLC sample disappears and then occurs. When the oil streak texture completely diminishes, the images become alternatively bright and dark when rotating the sample, indicating a transient nematic phase. Further, 450 nm light illumination made the oil streak texture reappear because the chirality inversion of HI-1 drives the formation of the CLC superstructure with opposite handedness. A similar texture disappearing and appearing could be observed by exposing the sample to 340 nm light, which was caused by chirality inversion of HI-1 during the conversion from PSS_450_ to PSS_340_. By alternately stimulating the sample with 450 nm and 340 nm light, the HTP of HI-1 changes reversibly for several cycles without obvious fatigue (Fig. 5d). Fig. 5c collates the HTP changes of the three hydrazone molecules HI-1, HI-2 and HI-3 in the initial, PSS_450_ and PSS_340_ states. It can be observed that all three hydrazone molecules have a chiral reversal ability.

**Fig. 6:**
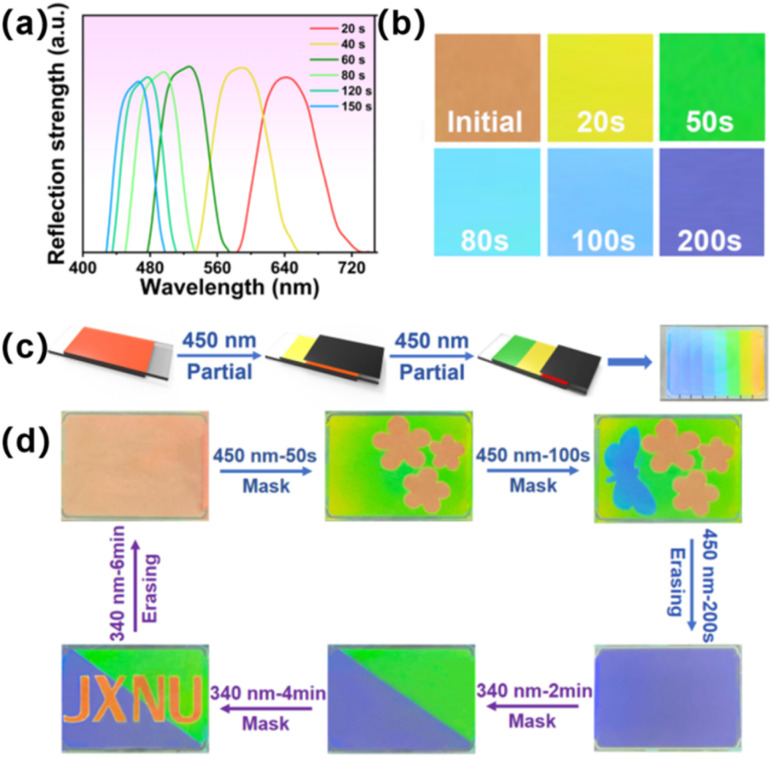
(a) Reflection band changes of CLC with 12 wt% HI-1 under 450 nm light. The light intensity is 1 mW cm^−2^. (b) POM images of the CLC sample after being exposed to 450 nm light in reflection mode. The light intensity is 1 mW cm^−2^. (c) Schematical illustration of the construction of a multi-color pattern by stepwise irradiating 450 nm light at different times using the photomask. On the right is the appearance of a rainbow-patterned CLC cell showing distinct visible colors. (d) Erasable and rewritable patterns in the CLC sample. The LC cell is 30 mm in length and 20 mm in width. The light intensity is 1 mW cm^−2^. All the vivid patterns were obtained under a left-handed polarizer.

To realize the dynamic modulation of visible light, 2 wt% chiral molecule S5011 and 12 wt% HI-1 were mixed with the LC matrix. After filling the sample into a planar cell, the prepared CLC presented a red color initially. As shown in Fig. 6a, the selective reflection band of the prepared CLC was centered at ∼641 nm. By irradiating the sample with 450 nm light, the reflection band of CLC blue-shifted to 486 nm within 80 s. When the HI-1 reaches PSS_450_, the reflection center moves to 460 nm in this case. Then it can be further modulated by 340 nm light irradiation. Fig. 6b is the corresponding POM images of the CLC sample in a reflection mode. A 450 nm light exposure led to a change of the CLC from red to green within 50 s, and then to blue within 100 s. Thanks to the superior thermal stability of the hydrazone switch, programmable visible patterns are constructible in the CLC cell by stepwise irradiating 450 nm light at different times using the photomask, as schematically illustrated in Fig. 6c. A rainbow pattern consisting of seven distinct visible colors was realized in a CLC cell by irradiating the sample at different times (Movie S1†). This provides an opportunity to fabricate erasable and rewritable display boards using remote light. For example, to obtain the RGB pattern, the CLC sample was first covered with a flower-sampled photomask and exposed to 450 nm light for 60 s. Then, the sample was covered with a butterfly-shape photomask and exposed to 450 nm light for 100 s. Due to the spatial photoisomerization of the HI-1 switch, the green background pattern and blue butterfly pattern can be written into the sample. By this method, different information could be stored, erased, and rewritten, like the pattern of the “JXNU” logo, as illustrated in Fig. 6d.

**Fig. 7:**
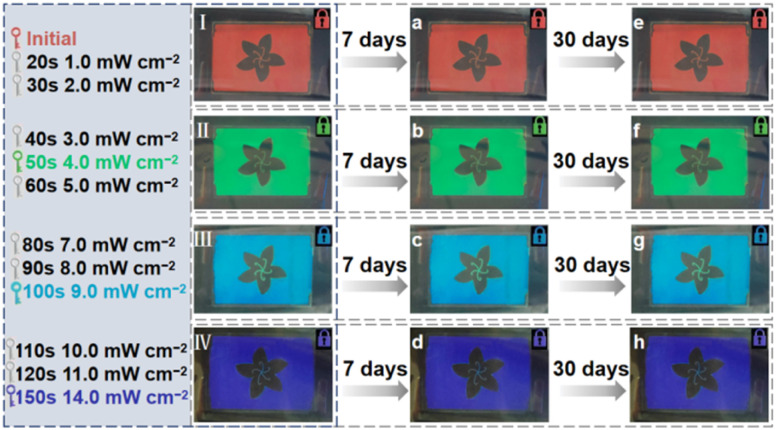
(I–IV) A flower-patterned CLC cell was obtained at different 450 nm light intensities. (a–h) The state of the flower-patterned CLC cell after 7 days and 30 days, respectively. All the vivid patterns were obtained under a left-handed polarizer.

To prove the accurate control and thermal stability of the light-driven chiral hydrazone switches, 2 wt% S5011 and 15 wt% HI-2 were mixed with 5CB. After filling the sample into an LC cell, the prepared CLC presents a bright red color initially. By controlling the irradiation intensity and exposure time of 450 nm light, an arbitrary structural color can be obtained, as shown in Fig. 7I–IV. Even after 30 days, the structural color of the CLC remains unchanged, indicating that the synthesized hydrazone switch has a high thermal stability, as illustrated in Fig. 7a–h. In short, the device can be used for color display by remote optical control and has an excellent thermal stability.

## Conclusions

In this work, a series of new chiral hydrazone switches, HI-1, HI-2 and HI-3, were designed and synthesized. Then, the photoresponsive behavior of the novel HI-1, HI-2 and HI-3 photoswitches were investigated. By incorporating the photoswitch into the LC host, light-driven CLC materials with handedness invertibility, photonic bandgap tunability, and a superior thermal stability were achieved. In addition, the stepwise regulation of light-driven CLC materials was also introduced. The direct application of CLC materials in erasable and rewritable display panels was demonstrated according to the optical drive thermal stability of the hydrazone switches. Although there have been reports of some other chiral invertible photoswitches,^46,47^ our novel HI-1, HI-2 and HI-3 photoswitches represent a type of molecular machine with chiral invertibility, large HTP changes, thermal stability, and negative photochromy (from light yellow to colorless), which is preferable in optical applications.

## Data availability

All data are available within the article and from the corresponding authors upon reasonable request.

## Author contributions

R. L. conceived the project and contributed to the design and analyses of the data. J. C. carried out most of the substance synthesis and experimental tests and wrote the draft of the paper. Z. W. carried out most of the calculations. Y. Y., J. H., S. Z. and H. Y. performed the experimental tests. X. C., T. D. and X. S. sorted out the picture layout. X. H. and X. Z. conducted the experiment. Z. S. guided the draft of the paper. All the authors discussed the results and commented on the manuscript.

## Conflicts of interest

The authors declare no competing interests.

## Supplementary Material

SC-OLF-D4SC05007J-s001

SC-OLF-D4SC05007J-s002
