# Supplementary material for: Dynamic handedness inversion of self-organized helical superstructures enabled by novel thermally stable light-driven chiral hydrazone switches
Source: Chem Sci. 2024 Sep 10;15(41):17041–8. doi: 10.1039/d4sc05007j (PMC11421039; doi:10.1039/d4sc05007j)
Supplement: SC-015-D4SC05007J-s002 [file SC-015-D4SC05007J-s002.pdf]

Supplementary Information (SI) for Chemical Science.
This journal is © The Royal Society of Chemistry 2024

# Supplementary Information

## Dynamic Handedness Inversion of Self-organized Helical Superstructures Enabled by Novel Thermally Stable Light-driven Chiral Hydrazone Switches

Jingyu Chen,[a] Zichen Wang,[b] Yuexin Yu,[a] Jun Huang,[a] Xinyu Chen,[a] Tongji Du,[a] Xinyue Song,[a] Haiyang Yuan,[a] Shuai Zhou,[a] Xiang-Guo Hu,[c] Xingping Zeng,[a] Shengliang Zhong *[a] and Ruochen Lan *[a]

[a] College of Chemistry and Materials, Jiangxi Normal University, Nanchang, 330022, China

[b] School of Materials Science and Engineering, Peking University, Beijing 100871, China

[c] National Research Centre for Carbohydrate Synthesis, Jiangxi Normal University, Nanchang, 330022, China

# Table of Contents

1. **Measurements ........................................................................................1**

2. **Synthesis.................................................................................................1**

3. **NMR characterization ..............................................................................3**

4. **UV-vis characterization...........................................................................8**

5. **HTP characterization ..............................................................................9**

6. **Band angle calculation..........................................................................10**

# 1. Measurements

$^{1}$H NMR spectra were recorded on a Bruker 400 MHz spectrometer. Chemical shifts (δ) are denoted in parts per million (ppm) relative to the reference tetramethylsilane (TMS) residual solvent peak ($^{1}$H δ = 0.00, $^{13}$C δ = 0.00; $CDCl_3$, $^{1}$H δ = 7.26, $^{13}$C δ = 77.16). The splitting patterns of peaks were designated as follows: s (singlet), d (doublet), t (triplet), m (multiplet), br (broad), or dd (doublet of doublets). ultraviolet/visible/near-infrared (UV/Vis/NIR) spectroscopy measurements were recorded using a PerkinElmer Lambda 950 spectrophotometer. The light (365 nm, 450 nm) irradiation was carried out with a LED lamp (FUV-6BK, Bangwo Elec. Technologies Co., Ltd., Guangzhou, China). Fluorescent properties of CLC were acquired using a Fluorescent spectrometer (PerkinElmer, LS55). The fluorescence images were taken under 365 nm light (0.1 mW $cm^{-2}$). Polarized optical micrographs were obtained using polarized optical microscopy (POM, Axio Scope A1 pol; Zeiss, Beijing, China). JASCO J-1500 instrument (Tokyo, Japan) was used to produce circular dichroism (CD) spectra, and CPL emission spectra were recorded on a JASCO CPL-300 (Tokyo, Japan).

# 2. Synthesis

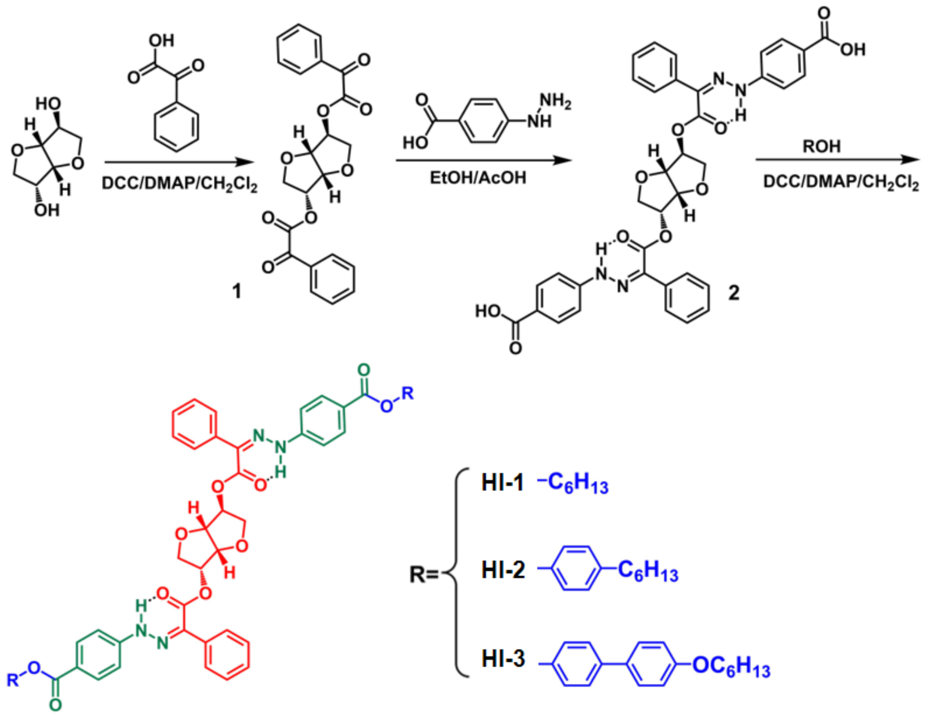

**1:** Benzoylformic acid (5 g, 33.3 mmol) and DMAP (0.74 g, 6.66 mol) was added to $CH_2Cl_2$ (100 ml). After the two solid was dissolved, S - isosorbide (2.21 g, 15.0 mmol) was added to them at 0 °C. After 10 minutes of reaction, add DCC (12.37, 600 mmol) to the mixture. The mixture was stirred while allowed to reach R.T. overnight. After removal of the solvent under vacuum, the product was purified by flash column chromatography (silica gel, EtOAc-PE=1:10→1:6) affording a yellow oil; $^1$H NMR (400 MHz, Chloroform-d) δ 8.09 (d, J = 7.4 Hz, 2H), 8.03–7.96 (m, 2H), 7.68 (dt, J = 7.6, 4.8 Hz, 2H), 7.53 (t, J = 7.7 Hz, 4H), 5.52–5.41 (m, 2H), 5.11 (t, J = 5.4 Hz, 1H), 4.68 (d, J = 5.0 Hz, 1H), 4.19–4.09 (m, 2H), 4.03 (ddd, J = 20.7, 10.9, 4.3 Hz, 2H).

**2:** A mixture consisting of **1** (3.04 g, 7.4 mmol) and 4-hydrazinobenzoic acid hydrochloride (2.48 g, 3.36 mmol) in ETOH (30ml) was heated to 80 °C and then four drops of AcOH was added to the solution. Stir for 4 h and let cool in the refrigerator overnight. The precipitate was collected by filtration yielding a yellow solid; $^1$H NMR (400 MHz, DMSO-d6) δ 12.54 (s, 2H), 11.64 (s, 1H), 11.47 (s, 1H), 7.91 (d, J = 8.4 Hz, 4H), 7.73 (d, J = 7.3 Hz, 2H), 7.67 (d, J = 7.0 Hz, 2H), 7.46–7.37 (m, 10H), 5.49 (q, J = 4.9 Hz, 1H), 5.39 (d, J = 3.2 Hz, 1H), 4.96 (t, J = 5.4 Hz, 1H), 4.69 (d, J = 4.9 Hz, 1H), 4.07 (d, J = 10.9 Hz, 1H), 3.98 (d, J = 4.5 Hz, 2H), 3.77 (dd, J = 11.2, 3.4 Hz, 1H).

**HI-1:** A mixture consisting of **2** (2 g, 2.94 mmol), hexoxy alcohol (0.66 g, 6.60 mmol), DCC (1.86 g, 9.00 mmol) and DMAP (0.11 g, 0.9 mmol) in $CH_2Cl_2$ at R.T. for 2 days. After removal of the solvent under vacuum, the product was purified by flash column chromatography (silica gel, EtOAc-PE=1:10→1:7) affording a yellow solid;$^1$H NMR (400 MHz, Chloroform-d) δ 12.20 (d, J = 87.8 Hz, 2H), 7.93 (d, J = 8.3 Hz, 4H), 7.56 (dd, J = 37.3, 7.3 Hz, 4H), 7.35–7.14 (m, 10H), 5.29 (dd, J = 10.8, 5.0 Hz, 2H), 4.87 (t, J = 5.3 Hz, 1H), 4.49 (d, J = 4.8 Hz, 1H), 4.20 (t, J = 6.7 Hz, 4H), 4.10–3.71 (m, 5H), 1.99–1.54 (m, 9H), 1.52–1.31 (m, 6H), 0.83 (d, J = 7.3 Hz, 6H). $^{13}$C NMR (101 MHz, Chloroform-d) δ 166.44, 162.39, 146.76, 146.54, 135.65, 135.57, 131.35, 129.64, 129.15, 128.58, 128.54, 128.27, 128.21, 128.08, 124.67, 124.40, 113.79, 113.66, 85.99, 81.04, 78.79, 74.79, 72.92, 71.03, 64.94, 33.98, 31.50, 31.46, 28.77, 25.75, 24.97, 22.57, 14.03.

**HI-2:** A mixture consisting of **2** (2 g, 2.94 mmol), 4-hexoxy-phenol (1.256 g, 6.60 mmol), DCC (1.86 g, 9.00 mmol) and DMAP (0.11 g, 0.9 mmol) in $CH_2Cl_2$ at R.T. for 2 days. After removal of the solvent under vacuum, the product was purified by flash column chromatography (silica gel, EtOAc-PE=1:10→1:7) affording a yellow solid; $^1$H NMR (400 MHz, Chloroform-d) δ 12.27 (d, J = 85.7 Hz, 2H), 8.08 (d, J = 8.3 Hz, 4H), 7.59 (ddd, J = 36.7, 7.7, 1.9 Hz, 4H), 7.41–7.23 (m, 10H), 7.03 (d, J = 9.0 Hz, 4H), 6.93–6.78 (m, 4H), 5.32 (dt, J = 7.9, 4.2 Hz, 2H), 4.90 (t, J = 5.3 Hz, 1H), 4.52 (d, J = 4.8 Hz, 1H), 3.89 (dq, J = 14.3, 7.8, 6.4 Hz, 6H), 1.71 (p, J = 6.8 Hz, 4H), 1.43–1.20 (m, 14H), 0.90–0.74 (m, 6H). $^{13}$C NMR (151 MHz, Chloroform-d) δ 165.27, 165.22, 162.38, 156.86, 147.32, 147.10, 144.42, 135.56, 135.49, 132.03, 130.12, 129.60, 128.61, 128.57, 128.40, 128.33, 128.13, 123.60, 122.47, 115.13, 113.92, 113.78, 85.98, 81.04, 78.86, 74.86, 72.92, 71.03, 68.48, 49.13, 33.97, 31.61, 31.46, 29.28, 25.74, 25.65, 24.97, 22.63, 14.05.

**HI-3:** A mixture consisting of **2** (2.5 g, 3.67 mmol), 4'-hexoxy-biphenol (2.18 g, 8.07 mmol), DCC (3.03 g, 11.01 mmol) and DMAP (0.18 g, 1.10 mmol) in $CH_2Cl_2$ at R.T. for 2 days. After removal of the solvent under vacuum, the product was purified by flash column chromatography (silica gel, EtOAc-PE=1:10→1:7) affording a yellow solid; $^1$H NMR (600 MHz, Chloroform-d) δ 12.25 (d, J = 135.1 Hz, 2H), 8.10 (d, J = 8.3 Hz, 4H), 7.63 (d, J = 7.5 Hz, 2H), 7.58–7.38 (m, 10H), 7.39–7.24 (m, 10H), 7.17 (d, J = 7.7 Hz, 4H), 6.89 (d, J = 8.3 Hz, 4H), 5.43–5.20 (m, 2H), 4.89 (t, J = 5.4 Hz, 1H), 4.51 (d, J = 4.9 Hz, 1H), 3.98–3.90 (m, 6H), 1.72 (p, J = 6.9 Hz, 4H), 1.61–1.31 (m, 10H), 1.10–0.94 (m, 4H), 0.91–0.72 (m, 6H). $^{13}$C NMR (151 MHz, Chloroform-d) δ 164.95, 162.39, 158.82, 150.04, 147.20, 138.62, 132.82, 132.12, 130.21, 129.69, 128.62, 128.57, 128.42, 128.36, 128.14, 128.12, 127.70, 121.99, 114.87, 113.96, 113.82, 85.98, 81.04, 78.87, 74.87, 72.93, 71.04, 68.16, 49.15, 33.97, 31.62, 29.29, 25.76, 25.65, 24.96, 22.63, 14.06.

# 3. NMR characterization

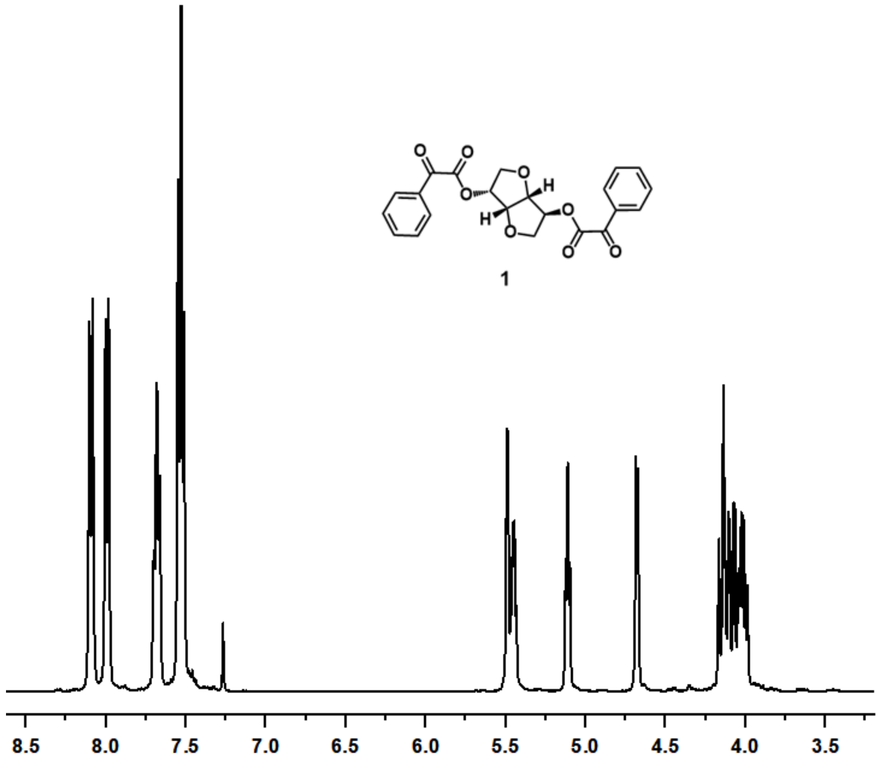


**Figure S1.** $^1$H NMR spectrum of **1** in $CDCl_3$ at 294 K.

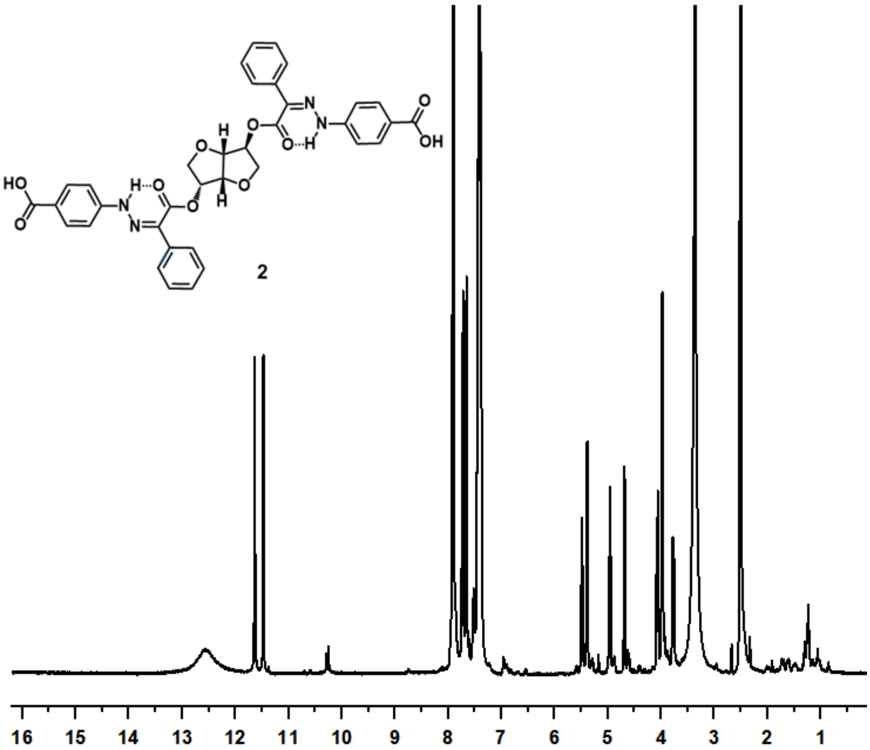


**Figure S2.** $^{1}$H NMR spectrum of **2** in DMSO at 294 K.

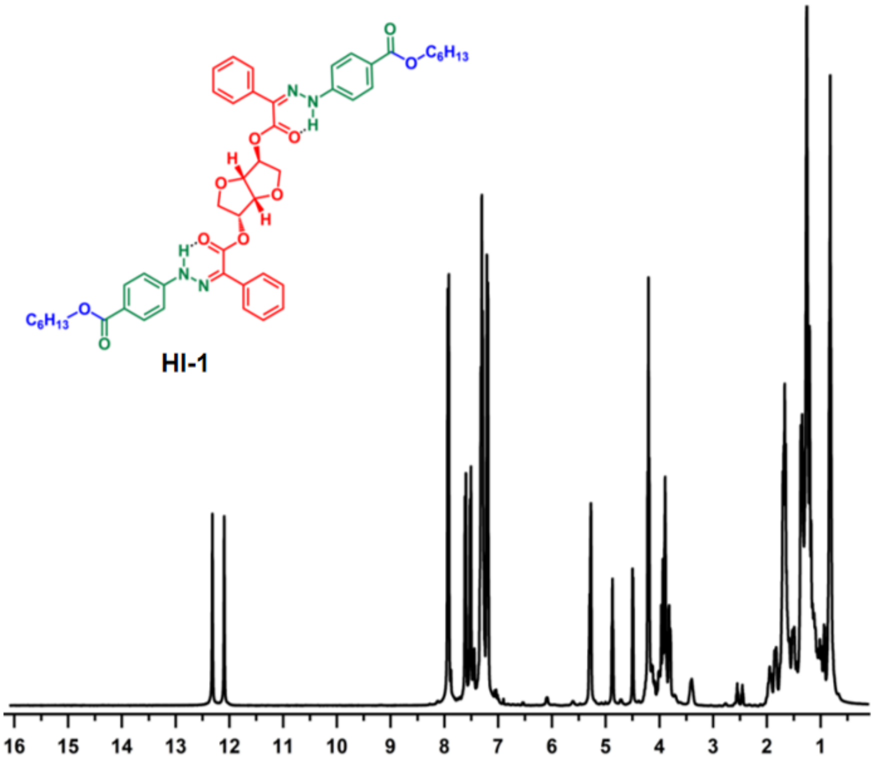


**Figure S3.** $^{1}$H NMR spectrum of **HI-1** in $CDCl_3$ at 294 K.

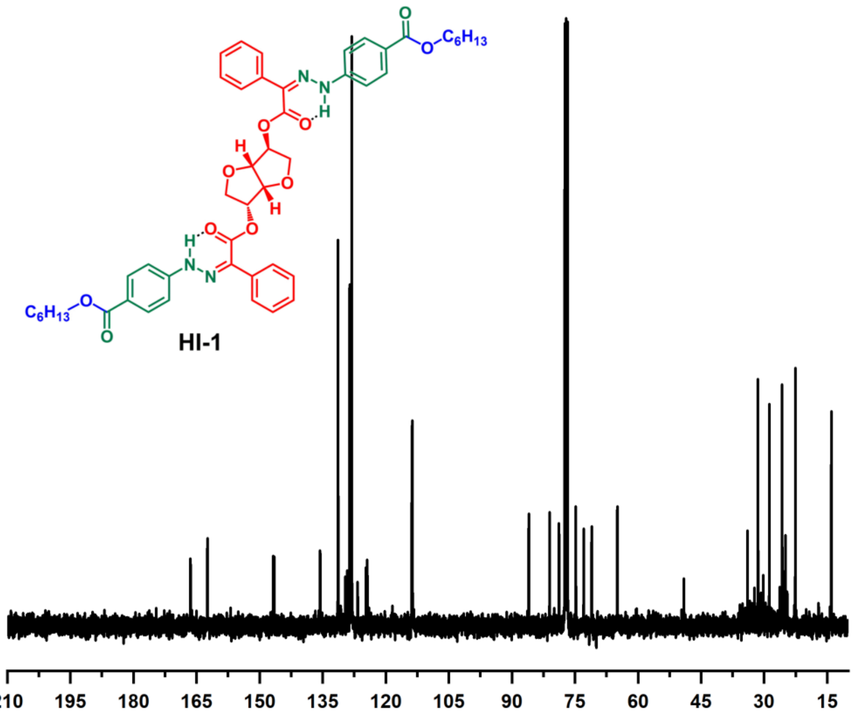


**Figure S4.** $^{13}$C NMR spectrum of **HI-1** in $CDCl_3$ at 294 K.

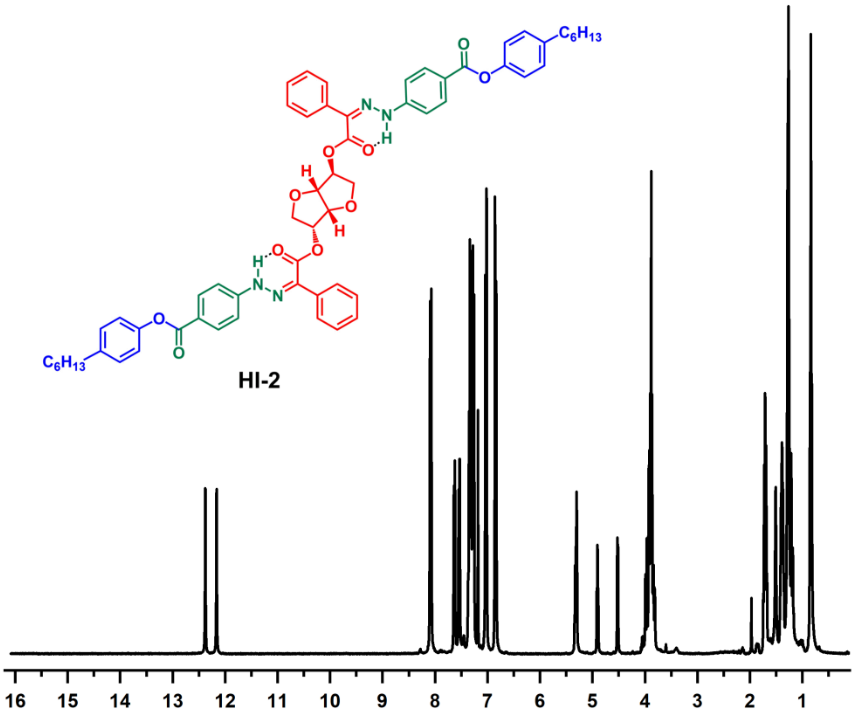


**Figure S5.** $^{1}$H NMR spectrum of **HI-2** in $CDCl_3$ at 294 K.

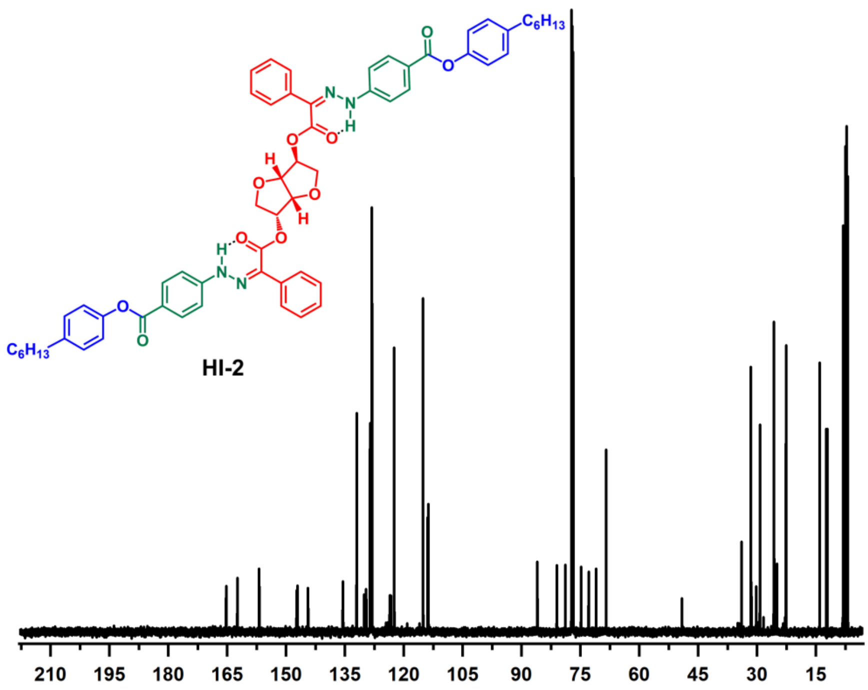


**Figure S6.** $^{13}C$ NMR spectrum of **HI-2** in $CDCl_3$ at 294 K.

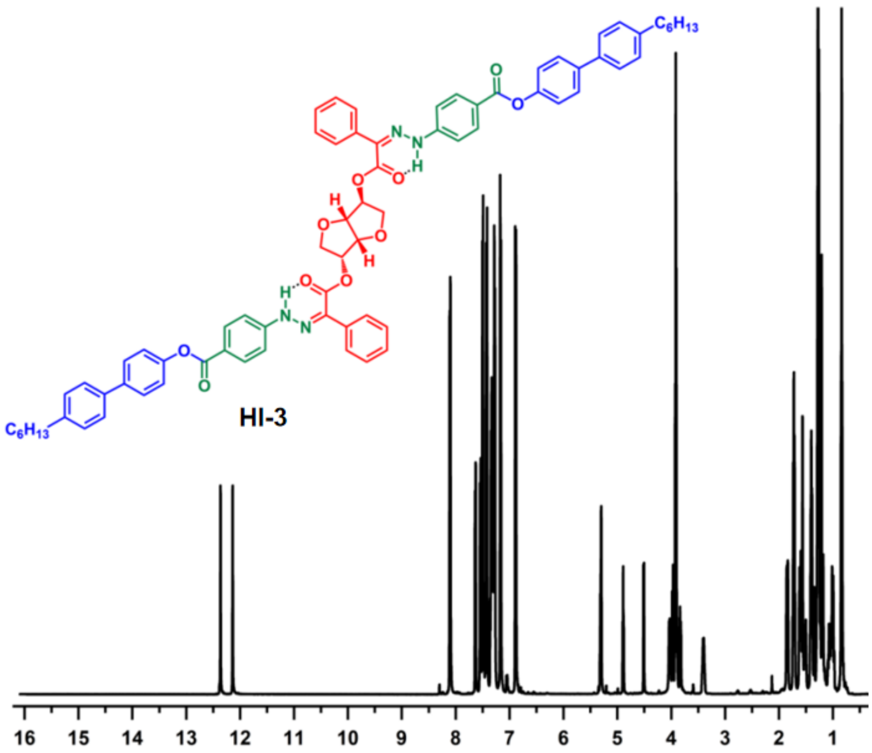


**Figure S7.** $^{1}H$ NMR spectrum of **HI-3** in $CDCl_3$ at 294 K.

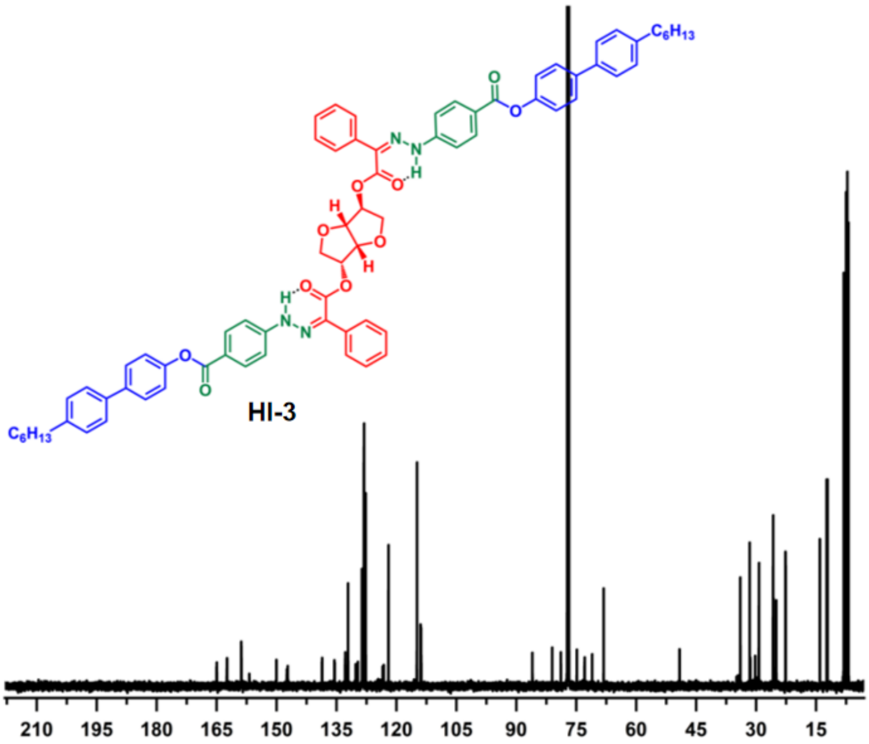


**Figure S8.** $^{13}C$ NMR spectrum of **HI-3** in $CDCl_3$ at 294 K.

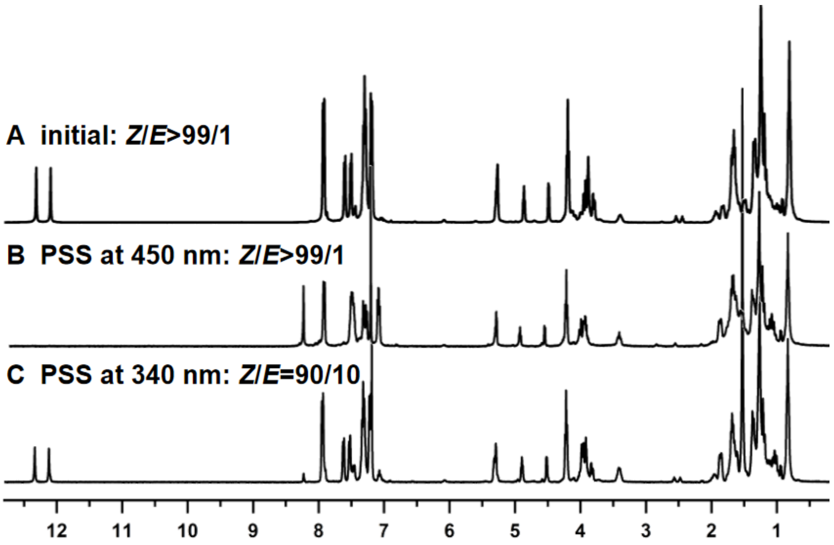


**Figure S9.** $^{1}H$ NMR spectra (Chloroform-d, 294 K) of **HI-1** (A) before, and after (B) 450 nm followed by (C) 340 nm photo irradiation to reach the PSS.

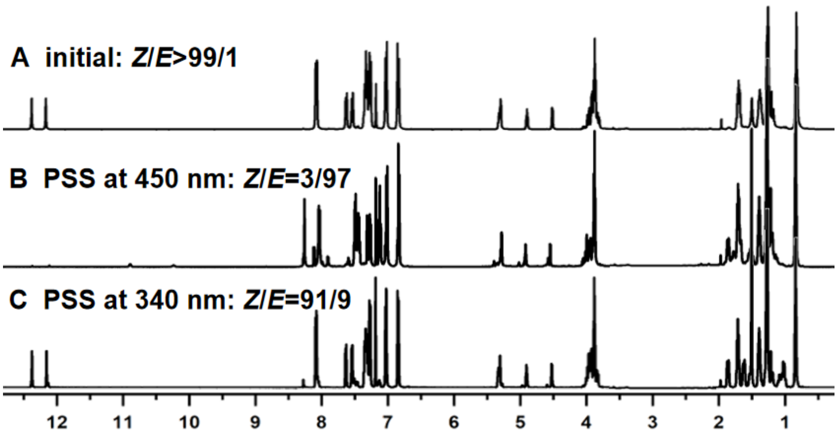


**Figure S10.** $^{1}$H NMR spectra (Chloroform-d, 294 K) of **HI-2** (A) before, and after (B) 450 nm followed by (C) 340 nm photo irradiation to reach the PSS.

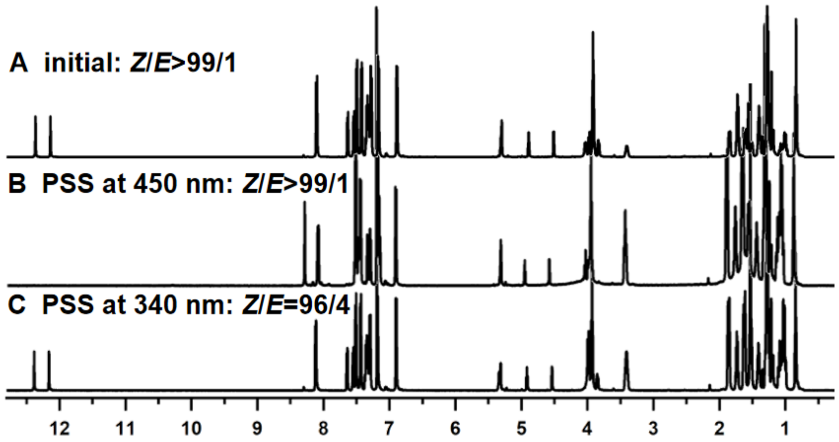


**Figure S11.** $^{1}$H NMR spectra (Chloroform-d, 294 K) of **HI-3** (A) before, and after (B) 450 nm followed by (C) 340 nm photo irradiation to reach the PSS.

# 4. UV-vis characterization

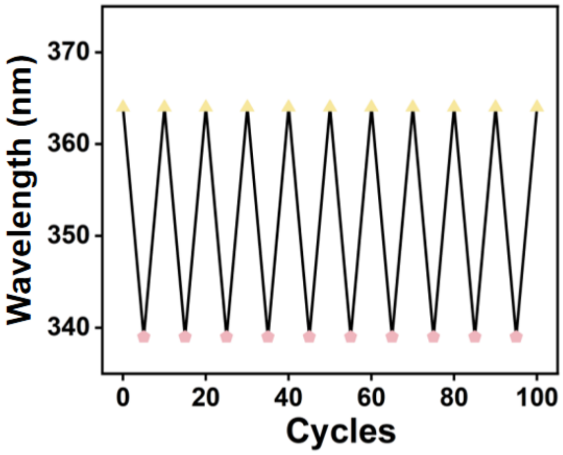


**Figure S12.** Cycling test of the photo-induced UV-vis changes of **HI-1**.

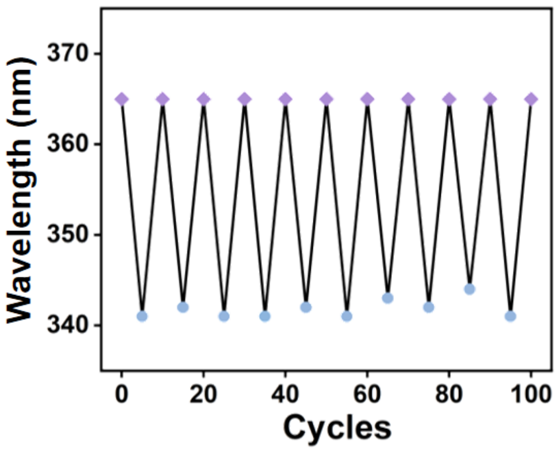


**Figure S13.** Cycling test of the photo-induced UV-vis changes of **HI-2**.

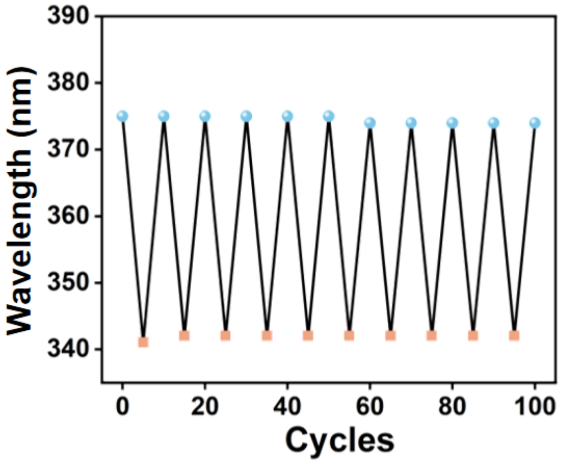


**Figure S14.** Cycling test of the photo-induced UV-vis changes of **HI-3**.

# 5. HTP characterization

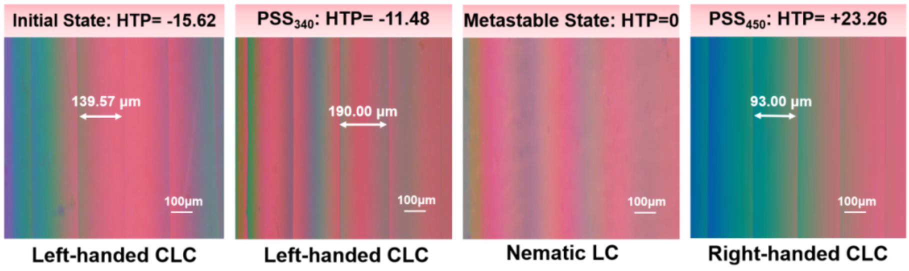


**Figure S15.** Fingerprint texture of the light-responsive **HI-2**-doped CLC in different states.

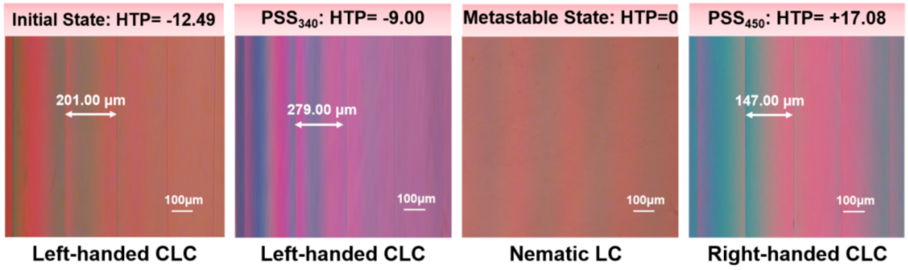


**Figure S16.** Fingerprint texture of the light-responsive **HI-3**-doped CLC in different states.

## 6. Band angle calculation

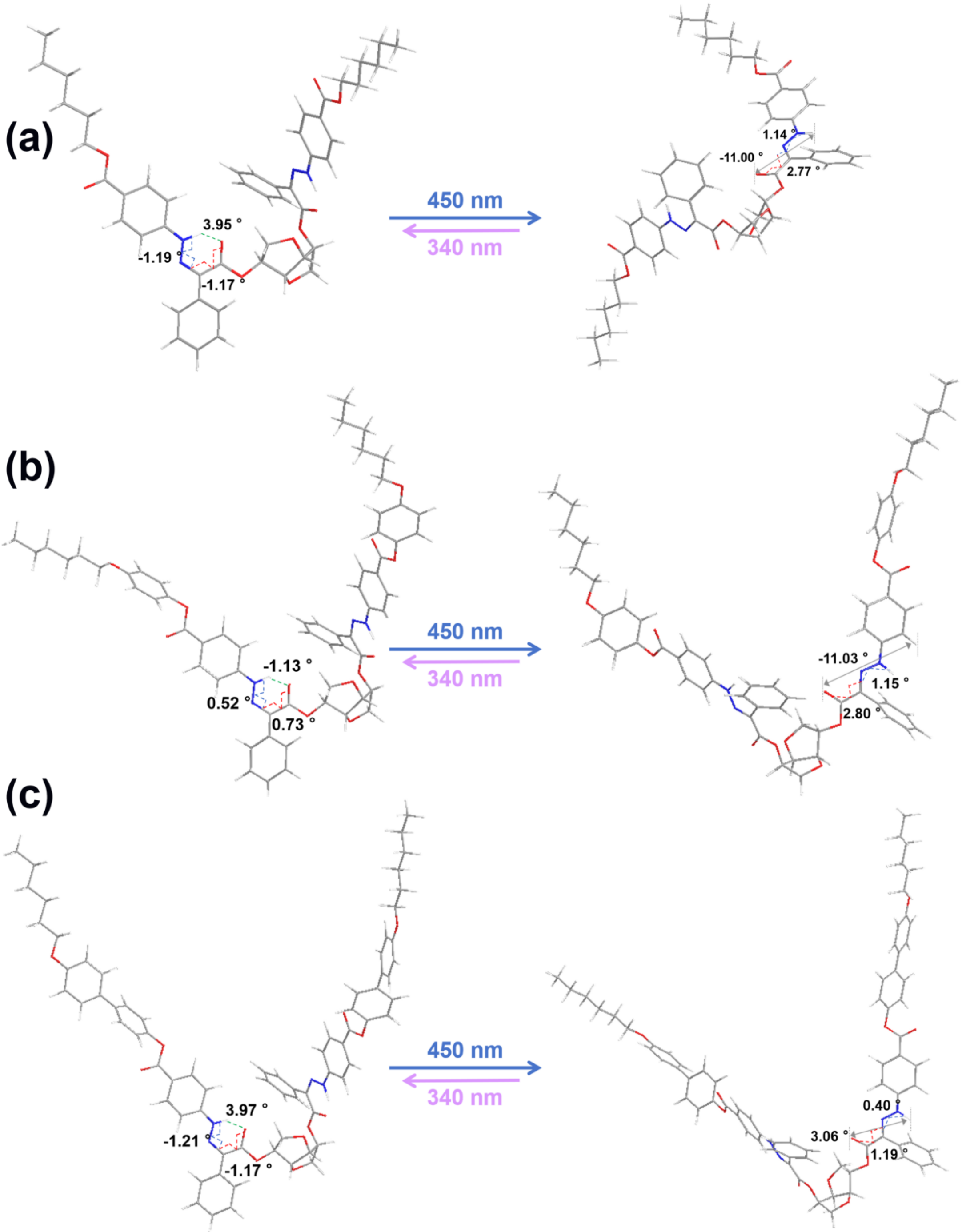


**Figure S17.** Crystal structures of **HI-1**, **HI-2**, and **HI-3** showed an intramolecular hydrogen bond (N–H···O) between the hydrazone NH proton and ester's C=O groups respectively (Figure 4).
